# Grape Winemaking By-Products: Current Valorization Strategies and Their Value as Source of Tannins with Applications in Food and Feed

**DOI:** 10.3390/molecules30132726

**Published:** 2025-06-25

**Authors:** Javier Echave, Antía G. Pereira, Ana O. S. Jorge, Paula Barciela, Rafael Nogueira-Marques, Ezgi N. Yuksek, María B. P. P. Oliveira, Lillian Barros, M. A. Prieto

**Affiliations:** 1 Nutrition and Food Group (NuFoG), Department of Analytical Chemistry and Food Science, Instituto de Agroecoloxía e Alimentación (IAA)—CITEXVI, Universidade de Vigo, 36310 Vigo, Spain; javier.echave@uvigo.es (J.E.); anolijorge@gmail.com (A.O.S.J.); paula.barciela@uvigo.es (P.B.); nogueirarafael29@gmail.com (R.N.-M.); ezginur.yuksek@uvigo.es (E.N.Y.); 2 CIMO, LA SusTEC, Instituto Politécnico de Bragança, Campus de Santa Apolónia, 5300-253 Bragança, Portugal; lillian@ipb.pt; 3 Investigaciones Agroalimentarias Research Group, Galicia Sur Health Research Institute (IIS Galicia Sur), SERGAS-UVIGO, 36312 Vigo, Spain; 4 REQUIMTE/LAQV, Department of Chemical Sciences, Faculty of Pharmacy, University of Porto, R. Jorge Viterbo Ferreira 228, 4050-313 Porto, Portugal; beatoliv@ff.up.pt

**Keywords:** grape waste, polyphenols, tannins, valorization, antioxidant, *Vitis vinifera*

## Abstract

Grape (*Vitis vinifera* L.) is one of the most extensively cultivated crops in temperate climates, with its primary fate being wine production, which is paired with a great generation of grape pomace (GP). GP contains a plethora of antioxidant phenolic compounds, being well-known for its high content of various tannins, liable for the astringency of this fruit. Winemaking produces a great mass of by-products that are rich in tannins. Grape seed (GSd) and pulp waste, as well as leaves and stems (GSt), are rich in condensed tannins (CTs), while its skin (GSk) contains more flavonols and phenolic acids. CTs are polymers of flavan-3-ols, and their antioxidant and anti-inflammatory properties are well-accounted for, being the subject of extensive research for various applications. CTs from the diverse fractions of grapefruit and grapevine share similar structures given their composition but diverge in their degree of polymerization, which can modulate their chemical interactions and may be present at around 30 to 80 mg/g, depending on the grape fraction. Thus, this prominent agroindustrial by-product, which is usually managed as raw animal feed or further fermented for liquor production, can be valorized as a source of tannins with high added value. The present review addresses current knowledge on tannin diversity in grapefruit and grapevine by-products, assessing the differences in composition, quantity, and degree of polymerization. Current knowledge of their reported bioactivities will be discussed, linking them to their current and potential applications in food and feed.

## 1. Introduction

Grape (*Vitis vinifera* L.) is one of the most extensively grown fruits in temperate climates, with more than 72 Mt of grapes produced in 2023, out of which more than 27 Mt of wine were produced globally, as estimated by the Food and Agriculture Organization [1]. Winemaking is indeed one of the oldest practices in human history, and wine itself is an intrinsic component of many cultures, especially in Mediterranean countries. In fact, European countries account for more than 60% of the world’s wine production, with Italy, France, and Spain being the top 3 producers globally. In Europe, 22.6 Mt of grapes were harvested, with 20.7 Mt being harvested for wine production only [2]. This great production of wine is paired with a similarly great volume of waste, as it is estimated that 20–30% of the grape weight input in winemaking ends up as grape pomace (GP), depending on the winemaking techniques and grape varieties, which includes grape skins (GSk), which can make up around 13–17% of the solid waste fraction, while seeds (GSd) account for 38–52% [3,4,5]. Additionally, a great mass of grape stems (GSt) are generated from grapevine pruning and management, representing approximately 2.5–7.5% of the total waste mass [6]. These facts have led to substantial research in the last decade revolving around grape waste management and revalorization strategies, often approached from a circular economy perspective in order to avoid landfilling. These winemaking by-products also contain high amounts of phenolic compounds, fibers, lipids, and colored pigments such as anthocyanins [7]. Despite its seasonality, GP and its individual components constitute a great mass of biomass waste, and various strategies have been developed to exploit it.

**Figure 1:**
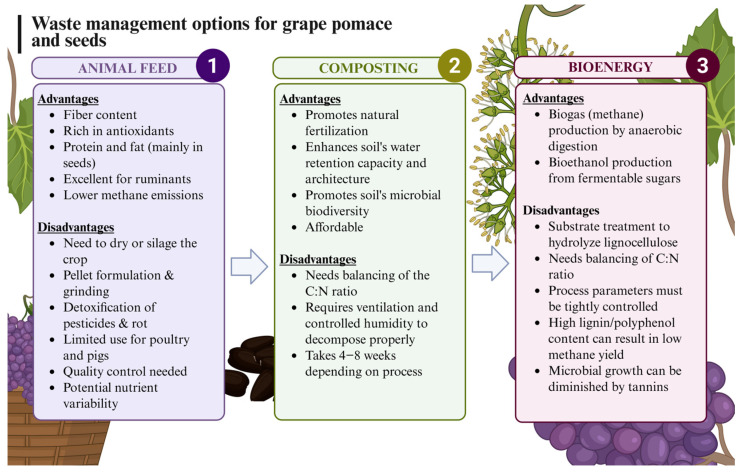
Current most extended grape pomace and seed waste management.

Some valorization approaches include its use as composting material, as it is rich in free sugars, fiber, and minerals, making it a notable component of organic matter in the soil. Its affordability and high fiber content make it ideal for composting. It is considered that GP has a good C–N ratio for composting; however, given its composition, high acidity, and possible influences due to growing conditions and varietal differences, some blending is usually needed [8]. Another alternative is its use as animal feed for these very same reasons [9]. On the other hand, GP may be used as a substrate for the production of biofuels following fermentation for the production of methane or ethanol or to be used as an organic source for biochar production with a high calorific value (Figure 1) [10,11]. Thus, various approaches with corresponding benefits and drawbacks are taken into consideration for GP valorization. Some of the current valorization strategies to valorize GP are also affected by the presence of tannins, of which GP is a rich source. Tannins are oligomeric and polymeric polyphenols with very significant antioxidant properties, as well as metal chelation, protein precipitation, and adhesive properties [12,13]. This provides these compounds with a variety of applications related to food and feed, especially in food processing or as fining agents due to their protein precipitation effects [14].

Thus, various other uses have been prompted by recent research for these wastes, such as the extraction and purification of various compounds in the nutraceutical and cosmetic industries, active packaging, or other uses in the food industry, such as the development of functional foods [15,16]. However, beyond these applications, tannins also account for various significant biological properties, such as antioxidant, antidiabetic, cardioprotective, antiobesity, and anti-inflammatory, among others [17]. For these reasons, tannins have been studied to be applied as functional ingredients in animal food and feed [18,19]. Specifically, as GP is a rich source of tannins, it has been reported that the direct use of GP in food and feed, as well as tannin-rich extracts, has promising applications in these fields by producing various benefits of interest for both humans and cattle [20].

The aim of this work is to present a comprehensive insight into *V. vinifera*’s GP as a significant source of tannins and discuss their chemistry as well as the various bioactive properties they can display and their extent. Current and future prospects for their use and application in food and feed will also be discussed.

## 2. Tannins: Structure and Considerations

Tannins are hydrophilic polymeric phenolics of molecular weight (mw) that range from 500 Da up to 30 kDa [21,22]. These polyphenols also appear as shiny or loose pale yellow or white powders with specific odors and a strong astringent flavor [23]. Moreover, they possess some properties not typical for any usual phenolic reaction, such as the precipitation of alkaloids, gelatin, and proteins [10,24,25]. Given their polymeric nature, tannins contain a large number of hydroxyl or other functional groups, which are found in the form of esters or hetero-esters and make them have a high binding affinity with various elements and molecules, especially metals and proteins [10,24,25]. Tannins in higher plants can be roughly divided into three structural groups: hydrolyzable tannins (HTs), condensed tannins (CTs), also called non-hydrolyzable tannins, and complex tannins, which combine monomeric precursors of HTs and CTs. The main distinction between HTs and CTs lies in the different monomers that serve as the polymer building block, which in turn also makes them more or less prone to hydrolyzation by acids or enzymes, with HTs being more easily susceptible than CTs [26,27,28]. HTs are mixtures of phenolic acids characterized by ester linkages connecting their primary chemical structures, usually to a glucose molecule. HTs range from 500 to 3000 Da, and their susceptibility to hydrolyzation facilitates their digestion and absorption in the digestive tract [18]. The phenolic acids that may polymerize into HTs are gallic acid and ellagic acid, which intuitively diverge into ellagitannins and gallotannins [29]. Upon hydrolysis, the ellagitannins are degraded to hexahydroxydiphenic acid, which then spontaneously forms ellagic acid by lactonization [21,30]. HTs are very common in tree barks (e.g., chestnut, oak, alder) and also in some fruit peels, with the most well-known example being pomegranate [31]. HTs can appear in aged wines due to the extended contact between the wine and the oak/chestnut aging barrels, allowing a transference from the barrel to the final product, conferring the wine a desirable astringency [32]. However, *Vitis vinifera* does not contain HTs, as only American *Vitis* species and their hybrids (e.g., *Vitis rotundifolia*) show HTs in their skins and, to a lower degree, in the seeds [33]. Although *V. vinifera* does not contain HTs, it is a very significant natural source of CTs [34].

CTs are oligomeric or polymeric flavonoids consisting of smaller fractions of polysaccharides and simple sugars and oligomers of flavan-3-ol units (catechins) and 3,4-flavan-diols (leucoanthocyanidins), forming oligomeric structures linked primarily by C4-C8 or occasionally C4-C6 bonds, with covalent bonds to catechin and epicatechin [12,18,35,36,37]. Four isomers, (+)-gallocatechin, (−)-epigallocatechin, (+)-catechin, and (−)-epicatechin, are formed due to the presence of asymmetric centers at the C-2 and C-3 positions of the monomeric units and conform the basic monomers of CTs [38]. Other variations include propelargonidins, with an mw of 578.52 g/mol, proteracacinidins (mw of 730 g/mol), and proguibourtinidins, which differ in the number of hydroxyl groups [29]. The monomer subunits in tannins are linked by either C4-C6 or C4-C8 linkages, although the C4-C6 linkage is more common in tannins where fisetinidin (resorcinol A-ring; catechol B-ring) and robinetinidin (resorcinol A-ring; pyrogallol B-ring) are the predominant repeated groups [39]. This is the typical conformation of B-type proanthocyanidins present in grapes, whereas A-type proanthocyanidins show a second linkage in the form of C2-*O*-C7 or C2-*O*-C5 [38]. Depending on the presence of galloylated isomers ((+)-gallocatechin, (−)-epigallocatechin) or catechins ((+)-catechin, (−)-epicatechin), CTs can be further classified into prodelphinidins or procyanidins, respectively [40]. As such, prodelphinidins are regarded as “proanthocyanin” CTs since they produce different anthocyanin precursors when depolymerized and also show a higher galloylation proportion [41]. Due to these facts, CTs present more complex structures and higher mw than HTs, typically in the range of 288 to >5000 Da [42]. CTs are more resistant to hydrolysis by heat, pH alterations, or enzymes, making them much more persistent in the environment, and their more ubiquitous distribution in plants and fruits makes them more common in diets [43,44]. CTs in *Vitis vinifera* may appear in the form of free monomers and in polymers of varied extension, usually up to 11 monomers. As the polymer size is related to a higher number of hydroxyl groups available for reaction and aromatic rings available for bonding and reaction, it has been observed that CTs of higher mean degree of polymerization (mDP) show a more pronounced reactivity, which is translated into a higher mouthfeel astringency [45]. This higher mw and reactivity allows them to form a higher number of bonds with macromolecules at different sites and precipitate them, as well as capture and precipitate metal ions, establishing bonds with higher affinity [44]. The most usual and studied CT polymers in grapes are usually the procyanidin dimers B1, B2, B3, and B4 and the procyanidin trimer C1, as they are usually the most abundant [46,47]. This is also due to the intrinsic difficulties in analyzing polymers of higher polymerization degree [46,47].

These binding properties are attributed to the chemical structures of tannins, which, as mentioned, contain two or three phenolic hydroxyl groups on a phenyl ring in moderately large molecules [19,48]. Moreover, the extraction and quantification method also plays a pivotal role in the accurate measurement of tannins, with some overestimations possible using spectrophotometric methods (acidified vanillin, phloroglucinolysis, protein, or methylcellulose precipitation) in contrast to chromatographic methods (quantification of specific oligomers and monomers) [49]. The quantification method can create great disparities in the measurement of CTs, with some methods, like the acidified vanillin method, potentially leading to an overestimation of the content, whereas protein precipitation can lead to an underestimation of the values [50,51]. The structures of CTs vary depending on the stereochemistry and hydroxylation pattern of the flavan-3-ol starter and extension units, the position and stereochemistry of the linkage to the “lower” unit, the degree of polymerization, and the presence or absence of modifications such as esterification of the 3-hydroxyl group [26]. The study of the physical and chemical properties of proanthocyanidins is an important area of research in oenology [30]. A notable and long-known feature of CTs is their influence on the astringency, color stability, and bitterness of wines. Factors such as DP and galloylation or subunit stereochemistry influence these sensations and features [23,52]. In addition, the mw and structure of CTs are generally found to determine their biological activities, with low-DP CTs having higher antioxidant activity, whereas high-CT DPs show enhanced protein and metal precipitation properties [42,49]. To our knowledge, there are no detailed explanations of the mechanisms involved in this feature, although molecular stability and possible oxidation/reduction synergies could be liable [53]. A proposed feature that may explain their different effectiveness in regard to DP is that higher DP CTs are less soluble than low DP CTs due to their mw, and there are various instances in which CTs with an mDP higher than 10 may show limited antioxidant activity in comparison with those of lower mDP [54]. Another possible explanation is that due to their smaller molecular size, CTs can interact more easily with key enzymes involved in the reported biological properties, as well as having more accessible hydroxyl groups, which are crucial for neutralizing free radicals. With increasing CTs’ molecular size, steric hindrance and intramolecular interactions can reduce the availability of these reactive sites, diminishing antioxidant potential [55]. On the other side, in some cases like enzymatic inhibition, CTs of higher DP may be more effective in enzymatic inhibition, especially of digestive enzymes like α-glucosidase, but those of lower DP have been repeatedly reported to be more effective in terms of antioxidant or anti-inflammatory activities [54]. This suggests that different bioactivities may peak at different polymer sizes, but antioxidant and anti-inflammatory effects were strongest at lower DPs. Conversely, with increasing DP, the ability to bind to small charged molecules like metals or molecules with a stronger charge like polysaccharides and their ease to form hydrogen bonds with them [56]. In the case of protein binding and precipitation, CTs usually bind with higher affinity to proteins rich in proline residues, a common feature in salivary proteins, thus better explaining their astringency [56]. Nonetheless, the methods that are tested to study biological properties (antioxidant, anti-inflammatory, antidiabetic) and their environment (in vitro, in vivo) and considerations on CT bioavailability and stability also influence the possible disparities in this regard [54].

## 3. Tannins in Grape By-Products

**Figure 2:**
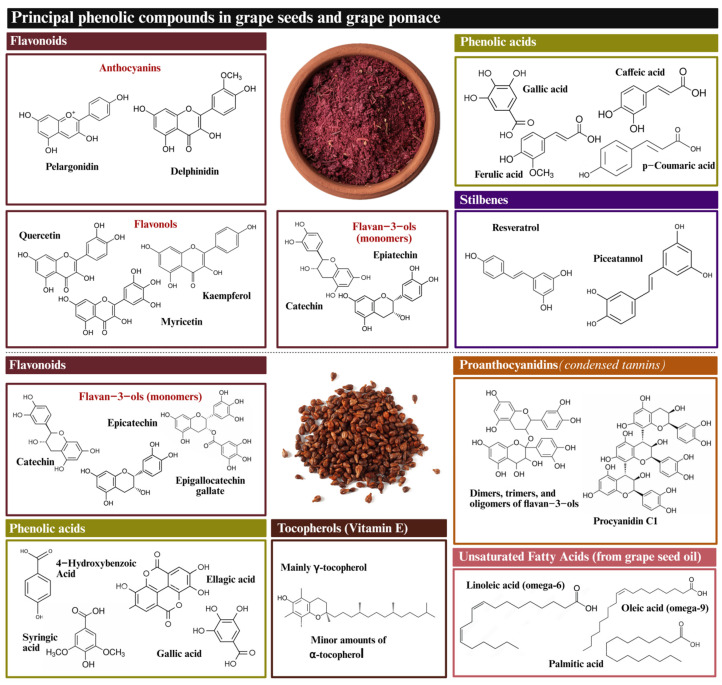
Chemical structure of whole grape pomace and grape seed major bioactive compounds.

GP and GSd are characterized as containing various amounts of polyphenols, including phenolic acids, flavonols, and stilbenes (Figure 2). Moreover, GSd is also well known to contain significant amounts of tocopherols and unsaturated fatty acids [57]. Yet, the most prominent element is CTs. Tannins are present in all grapevine by-products, including skins, seeds, and stems, with notable differences in their composition and concentration. These variations are influenced by the type of grape (red or white), the variety and/or cultivar, and the specific soil and cultivation conditions, which are also subject to annual variations. A detailed analysis of the tannin composition in each of these grape by-products with respect to their grape variety is presented in Table 1.

### 3.1. Skin

GSk, comprising approximately 5–10% of the total dry weight of the berry, typically contains significant concentrations of tannins [58]. However, the ratio of tannin to non-tannin phenolics varies among different grape cultivars, with flavonols and phenolic acids being the most prominent polyphenols present [46,50]. The major difference between red and white cultivars is that while red varieties present anthocyanins in their GSk, white grape cultivars usually show higher levels of non-colored polyphenols, especially phenolic acids, CTs, and also flavonols [59]. This is known to occur due to a lower flavan-3-ol synthesis by key enzymes such as leucoanthocyanidin reductase and anthocyanin reductase in favor of anthocyanin synthesis as the grape berry matures [45]. This also explains why these differences in CTs and other polyphenol contents between red and white varieties may not be so apparent in GSd, which usually displays more homogeneous levels of CTs among varieties [59]. The average concentration of tannins in the GSk is usually around 2 to 4 mg/g dw (Table 1). A wide study assessing the tannin content of 36 grape varieties by different methods available determined that the mean concentration of CTs in GSk could be around 4 mg/g fresh weight (fw), with the lowest values observed in the Malbec cultivar (1.7 mg/g fw) and the highest in table grape cultivars such as the Red Emperor cultivar (7.1 mg/g fw) [50]. Other reports indicate higher concentrations, up to 25 mg/g GSk in Cabernet Sauvignon, but this may be biased due to the CTs quantification method [60]. GSk are usually those of the highest mDP in all of the grape fractions, with variations between 8 and more than 20 monomers (Table 1). CTs in GSk usually present an mDP of around 8 to 9, with some exceptions (Table 1), and primarily include (+)-catechin, (−)-epicatechin, gallocatechin, and epigallocatechin, as well as oligomers of procyanidins and prodelphinidins [49,61]. In terms of composition, the main terminal monomer of GSk’s CTs is generally (+)-catechin, and (−)-epicatechin is the main extension subunit [62]. The proportions of each of these monomers have a strong varietal effect, with Cabernet Sauvignon, Merlot, and Syrah varieties standing out, for example, due to their high prodelphinidin content, more usual in these red varieties, as it is a precursor of anthocyanins [63]. Regardless of the variety analyzed, in most cases, these CTs are bound to other types of compounds, primarily polysaccharides, lipids, and proteins. This conformation means that tannins extracted from GSk have a chemical structure with a greater degree of branching and, therefore, a higher mw than tannins extracted from other parts of the vine [64]. These structures are modified during the grape ripening process, during which the degree of tannin polymerization increases [60]. This increased polymerization affects sensory analysis because it prevents these tannins from binding with salivary proteins, thereby reducing the level of astringency, which decreases with greater structural complexity of the CTs [65]. It has been observed that during grape ripening, the total amount of CTs varies significantly, reaching its highest levels around the time of veraison, with a subsequent decrease in their content afterward [66]. This decrease is attributed to enzymatic oxidation processes occurring after veraison.

**Table 1:** Condensed tannin composition, main degree of polymerization and concentration in *Vitis vinifera* grapefruit and grapevine.

Fraction	Cultivar	mDP	Units	Extraction Method	Concentration *	Ref.
**White grapes**						
Pomace	Chardonnay	4.5	Catechin, epicatechin, procyanidins B1, B2, B3, B4, procyanidin C1	PLE, Ace 80%, 103 bar, 5 min, 40 °C	71.9	[67]
	Macabeu	7.1			50.8	
	Parellada	5			92.1	
	Premsal Blanc	10.1			12.5	
	Sauvignon Blanc	na	Epicatechin	UAE, EtOH 60%, 300 W, 37 kHz, 20 min, S/L 1:30, 70 °C	23.8	[68]
	Pinot Gris	na			56	
	Gerwurztraiminer	na			58.2	
	Italian Riesling	na	Catechin, epicatechin	Maceration, acidified MeOH 50%, S/L 1:10, 6 h, 25 °C	0.68 mg/g fw	[69]
	Pinot Gris	3.4	Catechin, epicatechin	Maceration, EtOH 50%, S/L 1:10, 30 min, 40 °C	1	[70]
		2.7		PLE, EtOH 50%, 10.3 MPa, 10 min, 120 °C	3.4	
Skin	Malvasia bianca	26.4	Vanillin and phoroglucinol equivalents	Maceration, acidified MeOH 100%, 24 h, S/L 1:3, −20 °C Maceration, MeOH 80%, S/L 1:3, 4 h, 25 °C Maceration, MeOH 50%, S/L 1:3, 4 h, 25 °C	1.5	[51]
	Moscato bianco	25.6			1.1	
	Nascetta	24.9			0.72	
	Pinot bianco	34.4			0.9	
	Morio Muscat	na	na	UAE, acidified Ace 70%, S/L 1:4, 1 h, <45 °C	19.4	[71]
	Muller Thurgau				8	
	Moscato bianco	5.7	Epicatechin, catechin, epicatechin gallate, procyanidins B1, B2, B3, B4, procyanidin C1	UAE, EtOH 50%, S/L 1:10, 50 W, 20 min, 25 °C	3.1	[46]
	Arneis	6.6			5.3	
	Cortese	7.9			7.3	
	Garnacha	na	Catechin derivatives, polymeric procyanidins	Maceration, acidified MeOH 80%, S/L 12 h, 4 °C + UAE 5 min (3×)	1.3	[59]
	Maturana	na			1.5	
	Tempranillo	na			1.1	
	Viura	na			1.1	
Seed	Malvasia bianca	15.2	Vanillin and phoroglucinol equivalents	Maceration, MeOH 100%, 24 h, S/L 1:3, −20 °C Maceration, MeOH 80%, S/L 1:3, 4 h, 25 °C Maceration, MeOH 50%, S/L 1:3, 4 h, 25 °C	13.25	[51]
	Moscato bianco	11.5			13.35	
	Nascetta	10			5.47	
	Pinot bianco	9.4			9.5	
	Moscato bianco	4.4	Epicatechin, catechin, epicatechin gallate, procyanidins B1, B2, B3, B4, procyanidin C1	UAE, EtOH 50%, S/L 1:10, 50 W, 20 min, 25 °C	48.6	[46]
	Arneis	3.9			30.8	
	Cortese	6.6			57.9	
	Grenache	na	Catechin derivatives, polymeric procyanidins	Maceration, acidified MeOH 80%, S/L 12 h, 4 °C + UAE 5 min (3×)	11.8	[59]
	Maturana	na			13.2	
	Tempranillo	na			5.4	
	Viura	na			5.5	
	Riesling	na	Catechin, epicatechin, procyanidins B1, B2, B4	UAE, Ace 70%, S/L 2:15, 15 min, 50 °C (3×)	23	[72]
	Chardonnay	na			13.8	
Stem	Grenache	na	Catechin derivatives, polymeric procyanidins	Maceration, acidified MeOH 80%, S/L 12 h, 4 °C + UAE 5 min (3×)	6.1	[59]
	Maturana	na			8.6	
	Tempranillo	na			3.4	
	Viura	na			4.7	
	Airen	2.6	Catechin, epicatechin, gallocatechin, epicatechin gallate, prodelphinidin, procyanidins B1, B2, B4	MAE, EtOH 12.5%, S/L 1:5, 600 W, 12 min, 75 °C (3×)	1.2	[34]
**Red grapes**						
Pomace	Merlot	na	Epicatechin	UAE, EtOH 60%, 300 W, 37 kHz, 20 min, S/L 1:30, 70 °C	73.8	[68]
	Pinot Noir	na			79.9	
	Cabernet Sauvignon	na	Catechin	Maceration, EtOH 40%, S/L 1:50, 24 h, 25 °C	160 µg/mL	[73]
	Merlot	na			153 µg/mL	
	Terci	na			153 µg/mL	
	Cabernet Sauvignon	na	Catechin, epicatechin	Maceration, acidified MeOH 50%, S/L 1:10, 6 h, 25 °C	0.2 mg/g fw	[69]
	Merlot	na			0.04 mg/fw	
Skin	Cabernet Sauvignon	3.4	Catechin, epicatechin, gallocatechin, epigallocatechin, epicatechin gallate	Maceration, acidified EtOH 12%, 5 days, 30 °C	0.62	[61]
	Carmenere	2.1			0.93	
	Marzemino	2.6			1.1	
	Merlot	4.2			0.01	
	Pinot Noir	7			0.04	
	Shiraz	3.4			0.09	
	Teroldego	4.4			0.05	
	Cabernet Sauvignon	32.2	Vanillin and phoroglucinol equivalents	Maceration, MeOH 100%, 24 h, S/L 1:3, −20 °C Maceration, MeOH 80%, S/L 1:3, 4 h, 25 °C Maceration, MeOH 50%, S/L 1:3, 4 h, 25 °C	1.4	[51]
	Nebbiolo	42.5			1.9	
	Cabernet Sauvignon	4.1	Monomeric flavan-3-ols, dimeric procyanidins, catechin, epicatechin, epicatechin gallate	HAE, EtOH 10%, 2 h, S/L 1:200 20 °C	7.9	[74]
	Carménère	3.8			8.7	
	Grenache	na	Catechin derivatives, polymeric procyanidins	Maceration, acidified MeOH 80%, S/L 12 h, 4 °C + UAE 5 min (3×)	0.6	[59]
	Graciano	na			0.4	
	Maturana	na			0.04	
	Mazuelo	na			0.2	
	Tempranillo	na			0.3	
	Grenache	7.8–11	Catechin, epicatechin, procyanidin dimers and C1 trimer	Maceration, Ace 80%, S/L 6:55 4 h, 25 °C Maceration, MeOH 60%, S/L 6:55 4 h, 25 °C	35.2	[75]
	Shiraz	10.2–12.2			31.8	
	Carignan	10.3–12.1			46.2	
	Mourvedre	9.2–12.2			47.3	
	Counoise	11.2–11.7			37.6	
	Alicante	9.9–10.9			55.3	
	Barbera	4.4–6	Catechin, epicatechin, epicatechin gallate	UAE, EtOH 50%, 50 W, 48 kHz, S/L 1:10, 25 °C	7.5–21	[76]
Seeds	Shiraz	Proanthocyanidins	Catechin, epicatechin, epicatechin gallate	HAE. Ace 70%, 24 h, 1 mL, 0.1 g	0.06–0.09 mg/g fw	[77]
	Shiraz	Proanthocyanidins	Catechin, epicatechin, epicatechin gallate	HAE. Ace 70%, 50 mL, 24 h, 20 °C	25.9	[78]
	Grenache	2.6–14.6	Catechin, epicatechin, procyanidin dimers and C1 trimer	Maceration, Ace 80%, S/L 6:55 4 h, 25 °C Maceration MeOH 60%, S/L 6:55 4 h, 25 °C	83.1	[75]
	Shiraz	2.3–12			68.9	
	Carignan	3–10.4			78.8	
	Mourvedre	4.2–12.2			69.4	
	Counoise	2.5–11			70.9	
	Alicante	1.9–8.1			84.9	
	Cabernet Sauvignon	3	Catechin, epicatechin, gallocatechin, epigallocatechin, epicatechin gallate	Maceration, Acidified EtOH 12%, 5 days, 30 °C	57.7	[61]
	Carmenere	2.8			83.4	
	Marzemino	2			107.4	
	Merlot	3			72.7	
	Pinot Noir	3.2			317.1	
	Shiraz	3.5			63	
	Teroldego	3.9			148.3	
	Cabernet Sauvignon	11.2	Vanillin and phoroglucinol equivalents	Maceration, MeOh 100%, 24 h, S/L 1:3, −20 °C Maceration, MeOH (80%), S/L 1:3, 4 h, 25 °C Maceration, MeOH (50%), S/L 1:3, 4 h, 25 °C	12.1	[51]
	Nebbiolo	12.2			16.7	
	Grenache	na	Catechin derivatives, polymeric procyanidins	Maceration, acidified MeOH 80%, S/L 12 h, 4 °C + UAE 5 min (3×)	3.6	[59]
	Graciano	na			3.6	
	Maturana	na			1.7	
	Mazuelo	na			1.7	
	Tempranillo	na			2.2	
	Carménère	3.8	Monomeric flavan-3-ols, dimeric procyanidins, catechin, epicatechin, epicatechin gallate	HAE, acidified EtOH 10%, 2 h, S/L 1:200 20 °C	86.9	[74]
	Cabernet Sauvignon	4.3			90.3	
	Carménère	4.2	Catechin, epicatechin, procyanidins B1, B2, B4, B5, procyanidin C1, procyanidin B4-gallate	HAE, acidified EtOH (10%), 2 h, 20 °C	16.2	[79]
	Cabernet Sauvignon	4			11	
	Merlot	4.8			6.6	
	Cabernet Franc	5.2			8.2	
	Barbera	3.6–4.6	Catechin, epicatechin, epicatechin gallate	UAE, EtOH 50%, 50 W, 48 kHz, S/L 1:10, 25 °C	3.7–16.7	[76]
	Nebbiolo	4.2	Catechin, epicatechin, epicatechin gallate	UAE, Ace 50%, 50 W, 48 kHz, S/L 1:6, 20 min, 25 °C	8.3	[80]
Stem	Grenache	na	Catechin derivatives, polymeric procyanidins	Maceration, acidified MeOH 80%, S/L 12 h, 4 °C + UAE 5 min (3×)	4.8	[59]
	Graciano	na			4.5	
	Maturana	na			5.1	
	Mazuelo	na			3.5	
	Tempranillo	na			4	
	Cencibel	2.2	Catechin, epicatechin, gallocatechin, epicatechin gallate, prodelphinidin, procyanidins B1, B2, B4	MAE, EtOH 12.5%, S/L 1:5, 600 W, 12 min, 75 °C (3×)	1.3	[34]

Notes and abbreviations. * Units are expressed as mg/g dw unless stated otherwise. na: not analyzed; HAE: heat-assisted extraction; UAE: ultrasound-assisted extraction; MAE: microwave-assisted extraction; PLE: pressurized liquid extraction; MeOH: methanol; EtOH: ethanol; Ace: acetone; dw: dry weight; fw: fresh weight; mDP: mean degree of polymerization.

**Figure 3:**
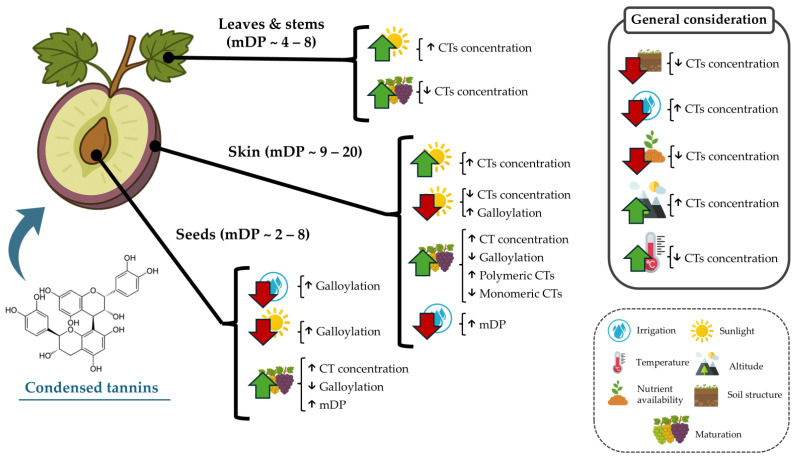
Environmental factors known to increase condensed tannin alterations in grape and grapevine fractions. Upward arrows depict increases; downward arrows indicate decreases or poorer quality.

Thus, GSk tannin concentrations may be indicated by increased sugar levels (°Brix) as maturation advances [50]. The concentration of tannins in GSk is also influenced by soil and climate factors, such as the amount of water supplied by rainfall or irrigation throughout the year, with drought conditions known to induce a higher synthesis of polyphenols in plants [38,81]. In the case of GSk, it has been reported that this can also induce a higher synthesis of polymeric CTs but does not reduce the levels of monomeric CTs in comparison with irrigated grapevines [60]. This was evidenced in Kékfrankos grapes subjected to different water availability conditions. It was observed that moderate water deficits during grape ripening caused an increase in the concentration of tannins in GSk and GSd of the analyzed grapes [82]. Another edaphoclimatic factor to consider is the growing region since significant differences were observed depending on the region analyzed, which seems to have a greater influence on the concentration and characterization of GSk tannins than the variety or seasonal variability [83]. In this sense, it is well known that the cultivation soil plays a pivotal role in the grape development and concentration of diverse components in the grape and the “terroir” of the developed wines. In this regard, as in the case of many other plant species, altitude also plays a key role in determining the tannin accumulation pattern, with a 500 m difference having been reported to increase CT concentration in Ekşikara GSk and GSd by at least 1.5-fold [81]. Sunlight exposure is also known to induce alterations in the CTs’ composition in GSk. It has been reported that a reduction in ultraviolet light induces a higher galloylation of CTs, with much higher proportions of epicatechin-3-gallate in the terminal subunits [62]. Poor nutrient availability and soil structure are also determinant factors that limit CT accumulation in both GSk and GSd [84]. Additionally, higher temperatures during maturation have also been consistently reported to decrease overall CT content in both GSk and GSd [85]. Thus, various factors beyond the grape variety and maturity stage affect the dynamics of CT accumulation in the grape (Figure 3).

### 3.2. Seeds

GSd constitutes approximately 6% of the berry weight and 25% in terms of mass of the by-products derived from grape cultivation [49,58,86]. GSd is characterized by having a higher concentration of CTs than pulp or GSk, usually containing as much as 60–70% of the total extractable phenolics in grapes, in comparison to 28–35% from GSk and pulp combined [38]. However, studies on other grape varieties have demonstrated that variations in tannin concentration can be even more pronounced, and seed tannin levels may differ as much as 289%, from around 157 to 455 mg/g dw [87]. As in the case of GSk, these proportions vary depending on the ripeness of the grapes, with higher CT content at the end of veraison [88]. Several studies have shown that most CT synthesis in GSd occurs after fruit set and is completed approximately five weeks before veraison, at which point the maximum concentration of proanthocyanidins is observed [74,86,89]. During grape ripening, CTs present in GSd tend to interact with proteins on the inner surface of the tonoplast, as well as with cell wall polysaccharides. These interactions reduce their bioavailability and extractability [90]. CTs present in GSd are typically composed of many fewer mDP units than in GSk at all stages of berry development [66], with mDP generally varying between 2 and 8 subunits (Table 1). However, in certain cases, such as Agiorgitiko grapes, GSd contained a higher number of monomeric units than GSk (8 vs. 2.8 mDP), which highlights the potential effects of grape cultivar on CT composition [91]. This lower degree of polymerization makes the tannins present in the GSd more reactive and, therefore, more prone to forming bonds with proteins, free radicals, and other molecules. Most of the tannins present in GSd are made up of three monomers (catechin, epicatechin, and epicatechin gallate) and procyanidin oligomers [61], procyanidins and prodelphinidins not having been detected, unlike in GSk [86]. Epicatechin constitutes approximately 65% of the extension subunits, making it the predominant component. Epicatechin-gallate comprises about 25%, whereas catechin contributes less than 10%, a proportion that is almost inversed in the case of GSk [49,51]. These relative proportions of extension subunits remained largely stable during berry development but with potential alterations at veraison, usually leading to a reduction in galloylation of the CTs and a slight increase in their mDP [38]. Regarding the influence of soil and climate factors on GSd tannin concentrations, further studies are needed, although it is noteworthy that although drought conditions may upregulate CT content and synthesis in GSd, the late stage of maturation and the cross-linking of these CTs with cell wall components can be translated into not significant increases of CT content, unlike the case of GSk (Figure 3) [89]. Currently, available studies are limited to evaluating water status (irrigation), with less significant differences observed in the content and structure of tannins present in GSd, unlike the effects observed in GSk [38]. The effect of temperature, altitude, sunlight exposure, soil nutrient availability, and structure has been reported to exert the same effects as in GSk [85]. However, conversely, to GSk, lower irrigation induces a marked increase in the galloylation of CTs due to a shift in the terminal subunit composition, while no other significant changes in terms of mDP have been observed [92]. Overall, it appears that GSd tannins are more resilient to abiotic factors than in the case of GSk.

### 3.3. Pulp

The grape pulp, or mesocarp, represents 80% of the berry mass, with the residual pulp after the winemaking process comprising 5–10% of the total GP [93,94]. This portion of the grape actually contains much lower levels of CT in comparison with GSk or GSd [95]. This significantly lower quantity of CTs is the reason why musts obtained from grapes without macerating the GSk or GSd do not exhibit the astringency or bitterness characteristic of wines [52]. Regarding the influence of soil and climate factors and the state of ripening on tannin concentration in pulp, there is considerable controversy, and no consensus has been reached on the mechanisms involved. Some studies conducted with varieties such as Pinot Noir or Shiraz observed a decrease in CT concentration over time, while in other varieties, such as Merlot or Tempranillo, an increase in their concentration was reported [96,97,98]. This hints at an apparent diversity in the grape variety CTs’ biosynthetic regulation. However, other studies report that the low concentration of CTs in this fraction of the grape remains relatively constant during the different stages of fruit ripening and sugar accumulation, with only a small increase in the degree of polymerization being observed over time [66,99]. These differences may be due to the use of different extraction techniques or differences in quantification methods [100]. This reaction ends at veraison, before the onset of anthocyanin biosynthesis. Therefore, the highest amounts of tannins per berry can be extracted during veraison, followed by a reduction in extractable tannins as the grape ripens [101,102]. In all cases, CTs are not considered to contribute significantly to the total phenolic content of the pulp or to the extractable potential in winemaking. Grape pulp, however, is rich in other types of phenolic compounds, particularly its high concentrations of simple phenolic acids and flavonols [103]. Among the tannins that can be identified, there are low levels of monomeric flavan-3-ols such as (+)-catechin or (−)-epicatechin [38,104]. The physiological function of these compounds in the pulp could be related to local oxidative defense mechanisms, although their biological role or potential application has not been widely studied since their levels in the grape flesh are low.

### 3.4. Prunings and Leaf Debris

During pruning, different plant parts are removed, the quantities of which may vary depending on the agricultural practice and the condition of the vine. Although exact data on the percentage of total waste generated by pruning is lacking, it is known that, along with fallen leaves, they constitute a significant portion of the vineyard’s residual biomass [105]. GSt, including leaves and stems derived from grape crushing during winemaking or as a result of pruning management, has only been explored as a source of polyphenols in recent years. Indeed, the literature indicates that lignocellulosic biomass derived from wine industry by-products contains a significant proportion of proanthocyanins. Specifically, grape GSts are reported to contain approximately 6% tannins [106,107], although in some cases CTs’ concentration can reach ≈17% [5]. These differences in concentration could be due to the difficulty in extracting these compounds from by-products, as they are usually found generally bound to lignans, compounds with a complex structure [108]. Regarding its composition, in a study carried out with the Spanish cultivars Airén and Cencibel, it was confirmed that there were no traces of HTs but a high proanthocyanidin content and an mDP close to 3 [34]. In both varieties, the most abundant fraction corresponded to CT dimers, mainly dimers B1, B2, and B4 (70–95%), followed by prodelphinidins (8–24%), a feature consistently observed in various varieties. It was observed that the concentration of the dimers decreased with storage time, while that of prodelphinidins increased, but at much lower levels (4–7%) than in GSk (~31%) [34,109]. In other cases, the reported CTs’ mDP may vary between 4 (Chardonnay) and 8 (Premsal Blanc), but in general terms, the CTs present are of a molecular size intermediate to those in GSk and GSd [110]. Likewise, composition is heavily influenced by grape varieties, with Chardonnay stems being reported to contain higher levels of (+)-catechin than procyanidin B1 [110]. These concentrations can be influenced by the type of management applied to the vineyard, such as pruning practices, as reported in a study carried out with Cabernet Sauvignon, in which it was observed that mechanical pruning reduced the concentration of CTs in the crop over time [111]. This suggests that proper management of these by-products could optimize their use to obtain high-added-value compounds. Yet, studies in grapevine content of CTs are still limited, but it has been reported that under drought conditions, the phenolic content of leaves and stems may be reduced, indicating a potential loss of CTs in these crops when approaching veraison [112]. Likewise, following maturation, CTs in stems have been described to decrease over time (Figure 3), reaching their minimum content at veraison [109].

## 4. Biological Properties of Tannins

### 4.1. Antioxidant

Oxidative stress is the result of normal metabolic reactions that result in the production of reactive oxygen species (ROS), which, if kept at sustained elevated levels, can devolve into chronic inflammation and cell death, which in turn loops a greater production of ROS. In this sense, the contribution of antioxidant phytochemicals, such as CTs, may prove a feasible strategy to limit oxidative stress [113,114]. CTs from grape by-products exhibit superior antioxidant activity compared with other compounds such as vitamin C and flavonoids [115].

Regarding their antioxidant mechanism of action, as in the case of other polyphenols, CTs form hydrogen bonds from the hydroxyl groups in their structure. This leads to the oxidation of the molecule and also to the stabilization of the phenolic rings in their structure [116]. This abundance of hydroxyl groups and their related antioxidant and binding activity is amplified by the degree of polymerization of the CT, which enables the compound to form complexes with oxidized forms of metals and other hydrophilic molecules, such as polysaccharides and proteins [117]. The antioxidant properties observed in in vitro models, including chemical assays with 2,2-diphenyl-1-picrylhydrazyl (DPPH) or thiobarbituric acid reactive substances (TBARS), are well-known [54]. In addition to direct chemical and bonding interactions, CTs are also capable of modulating the activity of various oxidizing enzymes present in foods and organisms, thereby preventing oxidation. One example of this is the inhibition of the enzyme xanthine oxidase and the improvement of the activity of endogenous antioxidant enzymes such as superoxide dismutase (SOD), catalase (CAT), or glutathione peroxidase (GSH-Px) [118]. Other evidence from studies conducted with animal models indicates that these extracts significantly reduce oxidative stress induced by heavy metals such as cadmium, arsenic, lead, and fluoride due to their metal-chelating properties as well as the enhanced expression and activity of the mentioned antioxidant enzymes [119]. Nonetheless, the exact mechanisms by which the interactions between metal ions binding to CTs work in regard to their action modes remain unknown despite their apparent effects [120]. In addition, as mentioned, although CTs usually display a higher antioxidant activity correlated with a higher mDP, this may be inversed when mDP exceeds 10, with mechanisms still largely unknown [54].

CT-rich extracts have also been reported to decrease oxidative stress biomarkers such as malondialdehyde (MDA) and nitric oxide (NO) [121,122]. As detailed below, this interlink between antioxidant and anti-inflammatory effects has also been evidenced in CTs being reported to prevent testicular [123], liver [124], and lung damage [125,126], as well as cell apoptosis in in vitro and in vivo models [119,121,127] (Table 2).

### 4.2. Anti-Inflammatory

The by-products derived from the processing of *Vitis vinifera* L. constitute a concentrated source of tannins with recognized bioactive properties, particularly in the modulation of inflammatory processes. Several studies have shown that these compounds exert anti-inflammatory effects through the regulation of key cellular mediators in the inflammatory response, both at the molecular and systemic levels (Table 2). Most of the available studies have been carried out with GSd, which has demonstrated in in vitro models a significant capacity to inhibit the activation of the nuclear factor (NF)-κB pathway, a crucial transcription factor in the expression of proinflammatory genes [154]. This inhibition results in decreased production of cytokines such as tumor necrosis factor (TNF)-α, interleukin (IL)-1β, and IL-6 in macrophages [155]. Additionally, a reduction in the expression of enzymes, such as iNOS and cyclooxygenase (COX)-2, involved in the synthesis of nitric oxide and proinflammatory prostaglandins, respectively, has been observed [156,157]. Various studies in animal models have shown that the administration of tannin-rich extracts from wine by-products reduces clinical markers of inflammation, such as edema and leukocyte infiltration (Table 2).

For example, CT-rich extracts have demonstrated inhibitory activity against proinflammatory molecules such as C-reactive protein (CRP), IL-6, and TNF-α, and the enhanced production of the anti-inflammatory cytokine adiponectin [132]. In another study, it was observed that this extract was able to exert a protective effect on Pb-induced lung injury due to the activation of the AMPK/Nrf2/p62 signaling pathway, thus providing a new approach for the treatment of Pb intoxication [127]. On the other hand, extracts rich in CTs have been shown to be effective in ameliorating diet-induced intestinal dysfunction and metabolic endotoxemia when administered at the end of a long-term obesogenic diet [125]. Moreover, CT-rich extracts have the potential to prevent and develop treatments for metabolic disorders by targeting gut microbiota [131].

These types of mechanisms had already been reported previously for CTs extracted from other plant matrices, such as raspberry fruits [158], and they are related to the modulation of oxidative stress, a phenomenon frequently associated with chronic inflammatory processes [159]. This dual action (antioxidant and anti-inflammatory) makes tannins bioactive agents of great interest for the management of chronic inflammatory conditions, such as cardiovascular and metabolic diseases [160]. In this sense, lower mDP tannins usually show more pronounced activities. For example, tannase-depolymerized CTs at a concentration of 200 μg/mL reduced IL-1β-induced secretion of PGE2 and IL-8 by 107% and 83%, respectively, and suppressed NF-κB activation by 63% [161].

These properties support their potential as therapeutic or nutraceutical agents in the management of inflammatory diseases and justify their inclusion in clinical trials aimed at validating their efficacy in humans.

### 4.3. Antidiabetic and Antiobesity

Type II diabetes and obesity are two highly prevalent and intertwined metabolic diseases currently considered to be on the rise globally, resulting in a high healthcare demand and associated costs. To prevent these pathologies, it is essential to implement lifestyle and dietary changes, and complementary alternatives based on naturally occurring bioactive compounds have been explored [162,163]. In this regard, CTs present in grape by-products have shown promising effects, and there is evidence suggesting their potential application as functional agents in the prevention or complementary treatment of metabolic disorders (Table 2).

The mechanisms of action of these compounds are very diverse. In the case of diabetes prevention, proanthocyanidin-rich extracts obtained from GSd have been shown to exert beneficial effects on glycemic homeostasis and oxidative stress [135,136,142]. Both factors are key to diabetes prevention, as they help maintain stable blood glucose levels and protect cells from damage associated with oxidative stress and derived inflammatory responses, thereby reducing the risk of metabolic disorders and insulin dysfunction [164]. Regarding the effective dose, there is no consensus in the available scientific literature. Relatively low doses (25 mg/kg body weight/day) showed a significant reduction in blood glucose levels in short periods (21 days) [136]. However, these effects were less pronounced over longer periods (41 days), in which no significant differences in glucose levels or insulin resistance were observed in studies conducted in rats, possibly due to an acquired resistance [137]. Higher doses (up to 500 mg/kg body weight/day) also significantly reduced blood glucose and improved pancreatic *β*-cell function [134]. In the case of CTs extracted from GSd, the beneficial effects on diabetes prevention were observed without significant differences attributable to dose [132,138]. Furthermore, CTs from GSd combined with metformin showed superior effects compared with individual treatments, suggesting a synergistic effect [135].

These extracts have also demonstrated beneficial effects on the control and treatment of obesity in animal models, acting mainly through appetite reduction and increased energy expenditure [126]. Doses between 350 and 1000 mg/kg body weight/day have been effective in acute and chronic treatments, associated with the modulation of the enteroendocrine system and the expression of GLP-1 [165]. Lower doses (25–30 mg/kg body weight/day) were effective in preventive treatments [136]. Furthermore, although weight loss was not identified in all study cases, reductions in fat mass and in the expression of adipogenic genes (Glut4, Srebp1c, Pparg2) were observed (Table 2). Therefore, the response varies in a dose-dependent manner, depending on the duration of treatment and the composition of the extract, which highlights the need to standardize protocols and establish safe limits.

Tannin extracts from grape residues have shown promising effects in the prevention and complementary treatment of obesity and diabetes by modulating adipogenesis, improving insulin sensitivity, and reducing oxidative stress. However, heterogeneity in doses, extract purity, and experimental models generates disparate results. Efficacy appears to depend on the initial metabolic status, duration of treatment, and synergy with other agents. Despite the positive effects observed, there is still no consensus regarding their long-term application. Standardizing protocols is essential to move toward robust clinical validation.

### 4.4. Cardioprotective

According to the World Health Organization, in 2019, there were more than 17.9 million deaths due to complications of cardiovascular diseases, representing 32% of all global deaths [166]. Cardiovascular diseases have various risk factors, one of the most significant being hypertension, which is influenced by habits such as diet, sedentary lifestyle, and tobacco and alcohol consumption [167]. In this context, diet plays a key role in the prevention and treatment of cardiovascular disease. Furthermore, CTs extracted from GP can be used in the development of nutraceuticals or dietary supplements with promising results [168,169]. Preclinical studies also support the use of these compounds in the treatment and prevention of cardiovascular disease. However, when analyzing the available evidence, several limitations and factors emerge that must be considered before proposing their use as an established clinical intervention.

GSd extracts show potential cardiovascular benefits, though results are mixed (Table 2). Some clinical trials report no significant blood pressure reduction in healthy individuals [170], but improvements are noted in those with cardiovascular risk factors [171,172,173]. These extracts can inhibit angiotensin-converting enzyme (ACE) and improve endothelial function through antioxidant effects, ameliorating cholesterol platelet deposition in blood vessels [174]. It may reduce LDL-c and total cholesterol, though high doses are often needed [129,175]. Animal studies demonstrate lipid-lowering and anti-inflammatory properties, with reduced oxidative stress and inflammatory markers like CRP [176]. However, human evidence remains limited, emphasizing the need for more rigorous clinical trials. In any case, the available results seem to show that the effects of grape tannin by-products are dependent on the physiological state of the individual (i.e., normotensive vs. hypertensive), the dose administered, the composition of the extract, and the duration of treatment. Furthermore, studies conducted with animal models seem to indicate that treatment with CTs could play a significant role as a therapeutic agent rather than as a preventive strategy [177], which limits their applicability to populations at risk or with a clinical diagnosis of hypertension. Other evidence from animal studies suggests that GSd-derived CT supplementation promotes white visceral adipose tissue distribution, which contributes to reducing the cardiometabolic risks associated with obesogenic diets [137]. Furthermore, a cardioprotective effect against diet-induced hypercholesterolemia has been observed due to its ability to reduce oxidative stress in the myocardium [136]. It also exhibits anti-hypertrophic and hyperplastic effects in white adipose tissue in rats with established obesity, hinting at a direct modulatory effect in key tissues [172].

Despite these advances in our understanding of the mechanism of action of the tannins in these by-products, the results observed in the literature are contradictory (Table 2). Numerous studies have demonstrated favorable outcomes associated with GSd extract; however, these investigations frequently utilize doses significantly higher than those typically obtained through a standard diet. Moreover, the current lack of well-designed clinical trials in human populations prevents concrete recommendations on its therapeutic use. Particularly notable is the loss of effect with prolonged treatment or the lack of response at high doses, which suggests the possibility of physiological adaptation or differences in the bioavailability and metabolism of the bioactive compounds. This variability highlights the urgent need to standardize both the composition of CT-rich extracts (proportion of flavanols, mDP, etc.) and administration protocols in experimental studies.

## 5. Current Food and Feed Applications of Tannins Derived from Grape By-Products

**Figure 4:**
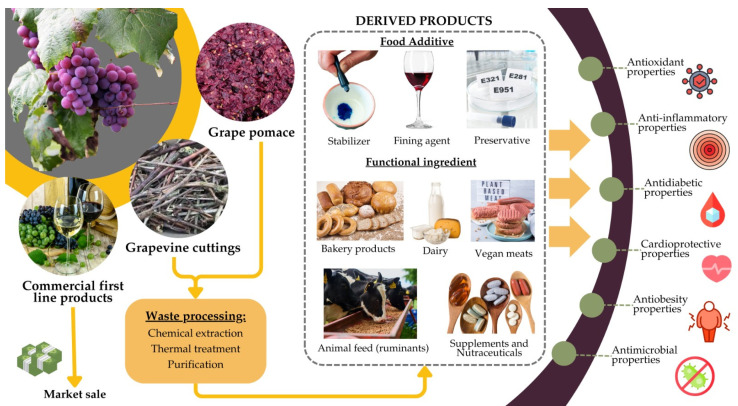
Depiction of alternative strategies of grapevine by-products’ valorization in food and feed due to their bioactive properties.

By repurposing grape by-products as sources of tannins, several food-related industries can enhance various products’ functional value, shelf life, and sensory attributes while promoting sustainability (Figure 4).

The utilization of grape by-products and derived CT-rich extracts as dietary supplementation in animal feed has been extensively studied, as it is a cheap addition to the feed, as, albeit being seasonal, it is an affordable ingredient. Furthermore, in recent years, GP feeding has been proven to provide additional benefits for livestock animals, especially ruminants [178]. For example, feeding lambs with grape seed tannin extract increased testis weight, volume, and antioxidant capacity, contributing to testis development and spermatogenesis [179]. GSd extract addition to goat and sheep feed at a concentration of 7.4.% for 11 weeks showed an increase in milk production in both animals, although it was more marked in goats, also yielding milk with lower lactose content [180]. Direct addition of GP at 20% to feed for lambs showed a significant increase in volatile fatty acids, while purine derivatives were decreased in the feces. At the same time, the lambs consumed fewer carbohydrates, and there were no differences in nitrogen utilization, showing that the added GP could improve feed utilization [181]. Another experiment with lambs revealed that adding GP at 10% to the feed for 74 days increased the meat’s antioxidant capacity and tenderness, while no other meat quality indicators or fat distribution were affected [182]. On the other hand, supplementation of cattle feed with GP has been reported to increase milk quality and production, generally by increasing the polyunsaturated fatty acid (PUFA) content in milk and lowering their atherogenic index [183]. This GP addition has also been shown to increase cattle immunological responses and lower oxidative stress markers, but a high consumption (20–30%) may also reduce weight gain in cattle [184]. In piglets, grape by-products can improve growth when added up to 9% of feed, and in the case of poultry, when added up to 3% of the feed [185]. Moreover, adding grape tannins to feed has enhanced meat quality and nutritional value in monogastric animals (pigs and poultry), usually by improving the PUFA content, tenderness, and color, a feature noted to be more pronounced with added GSd extracts [185].

Regarding functional feed applications beyond the improvement of meat, milk, and the animals’ oxidative status, it has been reported that feeding 2.5% condensed tannin extract to cattle fed a high-protein diet reduced ammonia emissions by 23%, explained by the scavenging of free ammonia by tannins in the rumen [186]. It has also been consistently observed that added GP limits ruminant’s methane emissions by limiting the growth of the animal’s methanogenic ruminal microbiota [187,188,189]. On the other hand, GP or GSd have also been shown to improve ruminants’ and piglets’ immunological response against various pathogens, suggesting that these grape residues may be a sustainable alternative to antibiotics in livestock disease management [183,190,191].

However, given their ability to form strong bonds with proteins and metals, CTs can also act as antinutritional elements, a property that is believed to be one of their ecological roles in plants [192]. Thus, a careful balance must be considered if GP, GSd, or other grape-derived elements are used as direct feed or extracts rich in CTs from these are added to the feed. For example, in the case of broilers, it has been observed that adding GP at a dose of more than 7.5% of the feed reduces feed intake in broilers [193]. In monogastric animals, high amounts of tannins can be particularly harmful as they are more prone to toxicity. Ruminants, due to their digestive system and microbiota, perceive fewer diminishing effects from GP- and CT-rich diets [184]. A notable mention is goats, which have been consistently reported to have tannin-binding salivary proteins that limit their reactivity through the intestinal tract, highlighting an evolutionary adaptation to tannin-rich diets [194]. There are still several gaps regarding the optimal addition in terms of content, which varies depending on the diet formulation (pellets, direct feeding), the added product (direct addition of processed by-product or extract), and the livestock species. Yet, in general terms, as extracts contain enriched fractions of CTs and other grape-derived polyphenols, a lower content in terms of mass in comparison with direct addition appears to be effective in inducing these benefits through feed addition [195].

One strategy to reduce potentially excessive CT content is to remove them with food-grade polyethylene glycol and ameliorate potential antinutritional effects [193]. Free CTs may also be partially removed by chemical extractions with food-grade solvents, as noted in Table 1, or following thermal treatments (~100 °C) of the substrate that induce the degradation of CTs and other components, such as cell walls [196] (Table 3).

On the other hand, the most common use for grape tannins is in beverages, mainly in wine, where they enhance wine’s flavor, stability, and color. Pre-fermentation addition of grape tannins also increases the varietal thiol content in wine, contributing to the tropical aroma and complexity, especially in varieties like Sauvignon Blanc [198]. Considering their protein precipitation properties, grape CTs have also been studied as fining agents to remove protein haze from wines [203]. Incorporating grape tannins into functional foods and dietary supplements adds a high antioxidant capacity and potential health benefits to these products, such as reducing oxidative stress and chronic inflammation [114].

Grape extract has also been used in dairy products such as Chobani’s^®^ Grape-flavored Greek yogurt and Stonyfield Organic’s^®^ Organic grape flavored Greek yogurt. Other common additions are grain bars (KIND Snacks^®^ KIND dark chocolate and sea salt healthy grain bars; That’s it^®^ Apple + Grape Fruit bars), cereal (Kashi^®^ GO LEAN crunch grape and green tea cereal; Bear Naked^®^ V’nilla almond granola), bakery products (Enjoy Life^®^ Grape soft baked cookies), and even candy (SunRidge Farms^®^ Grape Jelly Beans).

When applying grape extract or grape tannins to food products, their impact on food organoleptic properties must be considered. The most worrisome one is the astringency, which may not be desirable in all food products [204]. The addition of tannins can also lead to digestibility issues, as discussed in animal feed applications, and this problem also exists in humans. While grape tannins have strong antioxidant properties, their high mw can limit bioavailability and efficacy in some food applications, as they tend to form bonds with proteins and limit their breakdown and absorption. The polymeric nature of CTs in GSd extracts can affect their integration into food products, impacting their antioxidant and health benefits [205]. Indeed, added CTs can reduce the availability of digestible starch, as well as impair the action of α-amylase and α-glucosidase, limiting the absorption of glucose [206].

From a regulatory point of view, the use of tannins in the food industry is regulated by Communitarian and international legislations, like the FDA Code of Federal Regulations Title 21 and the Directive 2012/12/EU of the European Parliament and the Council, but limited recommendations are provided for the authentication and typification of raw materials [207]. Nonetheless, various grape-derived extracts have been submitted for evaluation by the European Food Safety Agency (EFSA), including tannin-rich extracts as well as crude extracts. In their reports, the EFSA states that to recognize tangible effects on human health, more abundant clinical evidence is needed [208], whereas its use as a flavoring agent in feed is approved and recognized [209]. In a recent EFSA evaluation involving the use of a commercial GP extract in poultry, its safety was ratified, but its efficacy as an antioxidant agent was not concluded, alluding to the need for further research, especially in standardized conditions [210].

## 6. Future Trends and Perspectives

As the food industry continues to evolve, there is an increasing interest in natural ingredients that improve the nutritional profile of food and meet consumer demand for sustainability and clean labels. Future trends in this area are expected to focus on innovative extraction techniques, novel applications in functional foods and nutraceuticals, and the development of environmentally friendly packaging solutions. Additionally, overcoming challenges such as astringency and bioavailability is essential for more widespread use of grape tannins.

Grape tannins have been suggested as an excellent addition to wellness drinks, a novel type of beverage specifically formulated to promote health and well-being. They typically contain ingredients that provide nutritional benefits, support physical and mental health, and enhance overall wellness. Reserveage^®^ Collagen Replenish Powder is a wellness product combining collagen with GSd extract.

Another novel use for grape extract is its application on food packaging, mainly biodegradable food packaging, providing preserving properties derived from its antioxidant power and bioactive compounds. GP extract can be used in tandem with natural fibers and shows potential as a new, bioactive food packaging material with high food preservation potential and antibacterial capacity against various foodborne bacteria [211]. Thermoplastic starch-based materials loaded with GSt extract also showed antifungal and antimicrobial properties, suggesting potential application in food packaging as active biomaterial layers [212]. Applying 0.5% GSd extract combined with a modified atmosphere in the packaging of roast chicken effectively reduces bacteria growth and lipid oxidation and maintains color stability during low-temperature storage, highlighting their use as preservatives [213]. Incorporating red grape extract in chitosan films showed similar results, improving the film’s properties, including antimicrobial activity, gas permeability, and swelling degree, contributing to sustainability and eco-friendliness in food/package production [214]. Thus, another feasible strategy can be incorporating grape extracts in functional food packaging or as an additive, presenting a promising advancement in enhancing food safety and extending shelf life. These natural compounds offer a sustainable alternative to synthetic additives, aligning with consumer demand for eco-friendly and health-conscious solutions.

Encapsulation is a novel and innovative technique that can mask the astringent flavor of grape extract. Encapsulation also potentially enhances the stability, bioavailability, and efficacy of the bioactive compounds found in grapes, protecting the sensitive bioactive compounds from environmental factors such as light, heat, and oxygen. Encapsulation also allows for controlled release and improved absorption in the body. Therefore, this process not only extends the shelf life of the active ingredients but also enhances their functional properties, making them more suitable for use in various foods [215].

For example, nanocapsules of grape and apple pomace phenolic extract in chitosan and soy protein enhance lipid solubility and antioxidant activity, with potential for use in edible food materials [216]. Another study encapsulated grape pomace polyphenolic extracts into mesoporous silica matrices, verifying reduced extract sensitivity and enhanced stability while maintaining their radical scavenger activity and cytocompatibility [217].

The encapsulated extract’s antioxidant power and properties also depend on the part of the grape that is used. Lavelli et al. suggest that encapsulated GSk phenolics have higher phenolic content, antioxidant activity, and inhibitory effectiveness against hyperglycemia damage than micronized GSk, with more extended storage stability and tailored food applications [218]. Spray-dried casein/pectin bioconjugate microcapsules loaded with GSk extracts significantly reduced phytochemical loss and created biocompatible and biodegradable products for nutraceutical applications [219].

Encapsulation can also be used to address the stability challenges of flavor substances. These techniques trap flavor substances in wall or carrier materials, which serve as physical barriers protecting the flavors from environmental factors. Recent studies suggest that nanoencapsulation may provide enhanced stability, encapsulation efficiency, and controlled release compared with microencapsulation due to the smaller particle sizes and increased surface area [220]. One of the main challenges in flavor encapsulation is preventing the diffusion of flavor substances through the encapsulation matrix, which can lead to off-flavors and reduced food acceptability. In the case of CTs, an unsuccessful encapsulation would lead to astringency, which the consumer does not desire. By addressing flavor volatility and stability challenges, encapsulation can improve functional foods’ sensory qualities and consumer acceptability [220].

## 7. Conclusions

In conclusion, condensed tannins derived from grape by-products represent a promising class of natural compounds with multifunctional applications in food and feed sectors. Their utilization aligns with principles of circular economy, waste valorization, and green chemistry, contributing to more sustainable agri–food systems. Despite the substantial progress made, challenges remain in terms of optimizing extraction techniques, standardizing bioactivity assessments, and ensuring regulatory compliance for their commercial use. Future research should focus on elucidating structure–function relationships, enhancing bioavailability, and evaluating long-term safety and efficacy in both human and animal models. With continued interdisciplinary efforts, CTs hold the potential to transform winemaking residues into valuable ingredients for health and sustainability.

## Figures and Tables

**Table 2:** Biological properties of grape seeds and derived extracts.

Source	Dose	Model	Results	Ref.
**Antioxidant**				
Seeds	PAE // 375 mg/kg bw	Wistar male rats	↓ oxidative stress markers, high blood pressure	[128]
Seeds	PAE // 100 mg/kg bw; 30 days	Wistar male rats	↑ antioxidant status; reverse of high lipid levels	[129]
Seeds	NE // 600 mg/day	Human	↑ plasma antioxidant capacity	[130]
**Anti-inflammatory**				
Seeds	PAE // 300 mg/kg bw/day, 7 weeks	C57BL/6 male mice	↓ TNF-α, IL-6, MCP-1	[131]
Seeds	PAE // 100–500 mg/kg bw/day, 2 weeks	Wistar female rats	↓ TNF-α secretions, transepithelial electrical resistance in small and large intestine, plasma LPS to basal levels	[125]
Seeds	PAE // 10 mL/kg bw/day, 3 weeks	Sprague-Dawley male rats	↓ myeloperoxidase, IL-1β, IL-6, TNF-α	[126]
Seeds	PCE // 345 mg/kg bw/day; 19 weeks	Zucker male rats	↓ CRP; ↑ adiponectin plasma levels; ≈IL-6 plasma levels	[132]
Seeds	PCE // 30 mg/kg bw/day; 15 weeks	Wistar female rats	↓ CRP, TNF-α, IL-6, Emr1; ↑ adiponectin in adipose tissue	[133]
Seeds	PCE // 200 mg/kg bw/day; 5 weeks	Wistar male rats	↓ inflammatory cells and TNF-α in lung tissue	[127]
**Antidiabetic**				
Seeds	PAE // 500 mg/kg bw/day; 16 weeks	Sprague-Dawley male rats	Partially reversed beta-cell dysfunction; ↑ insulin levels	[134]
Seeds	PAE // 100 mg/kg bw/day; 45 days	Wistar male rats	↓ glucose, insulin levels; ↑ insulin SE; restoration of the activities of glycolytic enzymes in the liver	[135]
Seeds	PAE // 25 mg/kg bw/day; 21 days	Wistar male rats	↓ glucose, insulin levels	[136]
Seeds	PAE // 25–200 mg/kg bw/day; 45 days	Wistar male rats	≈glucose, insulin, HOMA-IR plasma levels	[137]
Seeds	PCE // 345 mg/kg bw/day; 19 weeks	Zucker male rats	↓ glucose levels	[132]
Seeds	PCE // 25 mg/kg bw/day; 10–30 days	Wistar female rats	↓ insulin levels	[138]
Seeds	NE // 600 mg/day, 4 weeks	Human	Improvement of markers of inflammation and glycemia	[139]
Pomace	NE // 48.5 µg/mL	In vitro	↓ activity carbohydrate digesting enzymes	[140]
Pomace (red Portuguese varieties)	NE // 0.47 µg/mL	In vitro	↓ activity carbohydrate digesting enzymes	[141]
Pomace (Merlot)	NE // 50–250 mg/kg bw/day	Wistar male rats	↓ glucose levels	[142]
**Antiobesity**				
Seeds	PAE // 500 mg/kg bw/day; 8 days	Wistar male rats	↓ Food intake, bw; ↑ energy expenditure	[143]
Seeds	PAE // 25 mg/kg bw/day; 21 days	Wistar male rats	↓ adipocyte size; ≈bw, reversion of adiposity	[136]
Seeds	PAE // 100 mg/kg bw/day; 30 days	Wistar male rats	↓ bw	[129]
Seeds	PAE // 1000 mg/kg bw/day; 8 days	Wistar female rats	↓ Food intake, bw; ↑ energy expenditure	[144]
Seeds	PAE // 500 mg/kg bw/day; 14 days	Wistar female rats	↓ bw; ≈adiposity	[125]
Seeds	PAE // 500 mg/kg bw/day; 17 weeks	Wistar female rats	↓ bw, WAT, % visceral and total adiposity	[145]
Seeds	PAE // 300 mg/kg bw/day; 7 weeks	C57BL/6 male mice	↓ epidydimal fat mass; ≈bw	[131]
Seeds	PCE // 25 mg/kg bw/day; 15 days	Golden Syrian male hamsters	↓ adiposity, bw	[146]
Seeds	PCE // 25 mg/kg bw/day; 30 days	Wistar female rats	↓ visceral adipose tissue; ≈bw, plasma leptin levels	[138]
Seeds	PCE // 30 mg/kg bw/day; 15 weeks	Wistar female rats	↓ bw; ≈adiposity	[133]
Seeds	PCE // 345 mg/kg bw/day; 19 weeks	Zucker male rats	≈adiposity, bw	[132]
Pomace (Tannat)	NE // 2431.0 µg/mL	In vitro	Pancreatic lipase inhibition	[147]
**Cardioprotective**				
Seeds	PAE // 100 mg/kg bw/day; 30 days	Wistar male rats	↓ CHOL	[129]
Seeds	PAE // 25 mg/kg bw/day; 21 days	Wistar male rats	↓ CHOL	[136]
Seeds	PAE // 100 mg/kg bw; 10 weeks	Hamsters	↓ CHOL	[148]
Seeds (white grapes)	PCE // 375 mg/kg bw/day; 2 days	Spontaneously hypertensive male rats	↓ DBP, SBP	[149]
Seeds	PCE // 345 mg/kg bw/day; 19 weekss	Zucker male rats	↑ CRP; ↓ IL-6, TNF-α; ≈CHOL	[132]
Seeeds	NE // 0.5%; 10 weeks	Harlan Sprague-Dawley female rats	↓ arterial pressure	[150]
Seeds (Muscadine)	NE // 1300 mg/day, 4 weeks	Humans	↑ resting brachial diameter	[151]
Seeds	NE // 200–400 mg/day, 12 weeks	Human	↓ CHOL	[152]
Seeds	NE // 18.4 mg/kg bw; 12 weeks	Hamsters	↓ CHOL, atherosclerosis	[153]

Notes and abbreviations. NE: non-specified; bw: body weight; ↓: decrease; ↑: increase; ≈: no significant changes; SE: sensitivity; PAE: proanthocyanidins extract; PCE: procyanidins extract; IL: interleukin; LPS: lipopolysaccharides; TNF-α: tumor necrosis factor alpha; HOMA-IR: homeostasis assessment model for insulin resistance; WAT: total white adipose tissue; DBP: diastolic blood pressure; SBP: systolic blood pressure; CRP: C-reactive protein; CHOL: cholesterol levels.

**Table 3:** Valorization strategies of grape tannins and derived extracts.

Application	Description	Benefits	Example Products	Ref.
Animal Feed	Inclusion of grape tannins in animal diets to improve health and growth performance	Enhances gut health, antimicrobial properties, antioxidant effects, reduces methane production	Livestock feed, poultry feed	[179,185]
Food Preservation	Use of grape tannins as natural preservatives to extend shelf life and maintain quality of food products	Antimicrobial properties, antioxidant effects, extends shelf life	Packaged meats, dairy products, baked goods	[197]
Functional Foods	Addition of grape tannins to foods to enhance nutritional and functional properties	Antioxidant effects, potential health benefits (e.g., cardiovascular health, anti-inflammatory)	Nutraceuticals, fortified foods, beverages	[114]
Wine and Beverage Industry	Utilization of tannins in winemaking and other beverage formulations to enhance flavor and stability	Improves taste and aroma, stabilizes color, provides antioxidant benefits	Wine, juices, teas	[198]
Pet Food	Incorporation of grape tannins in pet food formulations to promote health and wellness	Supports digestive health, provides antioxidants, antimicrobial properties	Dog and cat food	[185]
Natural Colorants	Use of grape tannins as natural colorants in food products	Provides natural coloring, antioxidant properties	Confectionery, beverages, sauces	[199]
Dietary Supplements	Formulation of dietary supplements containing grape tannins for their health-promoting properties	Antioxidant effects, potential health benefits (e.g., cardiovascular health, anti-inflammatory)	Capsules, powders, tablets	[114]
Meat Products	Application of grape tannins in meat processing to improve quality and shelf life	Antimicrobial properties, enhances flavor, antioxidant effects	Sausages, cured meats	[200]
Bakery Products	Incorporation of grape tannins in bakery products for enhanced nutritional value and shelf life	Antioxidant properties, potential health benefits, extends shelf life	Bread, cakes, pastries	[201]
Dairy Products	Use of grape tannins in dairy processing to enhance nutritional and functional properties	Antioxidant effects, potential health benefits, improves texture and flavor	Yogurt, cheese, milk-based drinks	[202]

## Data Availability

No new data were created or analyzed in this study. Data sharing is not applicable to this article.

## Abbreviations

The following abbreviations are used in this manuscript:

GP	Grape pomace
GSk	Grape skins
GSd	Grape seeds
GSt	Grape stems
CTs	Condensed tannins
HTs	Hydrolyzable tannins
mDP	Mean degree of polymerization
fw	Fresh weight
dw	Dry weight
SOD	Superoxide dismutase
CAT	Catalase
GSH	Glutathione
MDA	Malondialdehyde
TBARS	Thiobarbituric acid reactive substances
NO	Nitric oxide
NF-κB	Nuclear factor kappa B
IL	Interleukin
TNF-α	Tumor necrosis factor-alpha
iNOS	Inducible nitric oxide synthase
COX	Cyclooxygenase
CRP	C-reactive protein
AMPK	Amp-activated protein kinase
PPAR	Peroxisome proliferator-activated receptor
PGE	Prostaglandin E
GLP	Glucagon-like peptide
LDL	Low-density lipoprotein
